# Interplays of *ADH1B* Genotype, Alcohol Consumption, and Gut Microbiota in Relation to Insulin Resistance

**DOI:** 10.3390/nu17162669

**Published:** 2025-08-18

**Authors:** Brian Wang, Brandilyn A. Peters-Samuelson, Kai Luo, Christina Cordero, Krista M. Perreira, Amber Pirzada, Martha L. Daviglus, Yang Li, Robert C. Kaplan, Robert D. Burk, Qibin Qi

**Affiliations:** 1 Department of Epidemiology and Population Health, Albert Einstein College of Medicine, Bronx, NY 10461, USA; brian.wang@einsteinmed.edu (B.W.); brandilyn.peterssamuelson@einsteinmed.edu (B.A.P.-S.); kai.luo@einsteinmed.edu (K.L.); robert.kaplan@einsteinmed.edu (R.C.K.); 2 Department of Psychology, University of Miami, Miami, FL 33124, USA; c.cordero@umiami.edu; 3 Department of Social Medicine, University of North Carolina, Chapel Hill, NC 27599, USA; perreira@email.unc.edu; 4 Institute for Minority Health Research, University of Illinois at Chicago, Chicago, IL 60612, USA; apirza2@uic.edu; 5 Department of Preventive Medicine, University of Illinois at Chicago, Chicago, IL 60612, USA; daviglus@uic.edu; 6 Department of Medicine and Population Health, NYU Grossman School of Medicine, New York, NY 10016, USA; yang.li@nyulangone.org; 7 Department of Pediatrics, Albert Einstein College of Medicine, Bronx, NY 10461, USA; robert.burk@einsteinmed.edu

**Keywords:** ADH1B genotype, alcohol intake, insulin resistance, interaction, gut microbiome

## Abstract

Background/Objective: Alcohol consumption has been linked to alterations in gut microbiota and insulin resistance. The alcohol dehydrogenase 1B (ADH1B) gene plays a crucial role in alcohol catabolism, where rs1229984 variant carriers (CT/TT) catabolize ethanol at an 80-fold faster rate than non-carriers (CC). This study investigates the relationships between ADH1B gene rs1229984 mutation, alcohol consumption, gut microbiota, and insulin resistance. Methods: We performed cross-sectional analysis on fecal metagenomic sequencing data from diabetes-free participants in a longitudinal cohort of the Hispanic Community Health Study/Study of Latinos. We used Analysis of Composition of Microbiomes to identify gut microbial species associated with alcohol consumption in non-carriers (*n* = 1399) and carriers (*n* = 193). We constructed genotype-specific gut microbiome scores (GMSs) based on the identified species associated with alcohol consumption to examine how gut microbiota may influence the relationship between alcohol consumption and insulin resistance across *ADH1B* genotypes. Insulin resistance was defined as Homeostatic Model Assessment for Insulin Resistance (HOMA-IR) > 2.5. Results: Distinct microbial species associated with alcohol consumption were identified in non-carriers (54 species) and carriers (16 species). In non-carriers, the genotype-specific GMS modified the relationship between alcohol consumption and insulin resistance (P_interaction_ = 0.011). The odds ratios (OR) for insulin resistance with increasing alcohol consumption levels across low, moderate, and high tertiles of GMS were 0.75 (95%CI 0.58–0.96), 0.82 (0.67–1), and 1.13 (0.93–1.39), respectively. We identified that individual alcohol-related species, such as Prevotella copri, Ruminococcus callidus, and Erysipelatoclostridium ramosum, modified the relationship between alcohol consumption and insulin resistance in non-carriers. Conclusions: This study suggests that the ADH1B gene rs1229984 mutation is associated with gut microbiota profiles altered by alcohol consumption. Our findings also suggest a potential role of gut microbiota in the protective association between alcohol consumption and insulin resistance in the *ADH1B* variant non-carriers.

## 1. Introduction

Insulin resistance is a major risk factor for type 2 diabetes [1], affecting more than 1/10 of adults in the US [2]. Individuals with diabetes face a twofold excess risk for cardiovascular disease (CVD), the leading cause of death in the US [3]. Notably, insulin resistance is also linked to the development of cardiometabolic complications, with risk already arising prior to the onset of type 2 diabetes [4,5]. Prior research has greatly advanced our understandings on the risk factors for insulin resistance, including its genetic basis [6] and lifestyle factors, such as diet, exercise, smoking, sleep, and stress [7]. Epidemiological studies have suggested a potential link between alcohol consumption and improved insulin sensitivity; however, findings have been inconsistent across studies. While some studies reported that alcohol intake was associated with improved insulin sensitivity and reduced risk of type 2 diabetes [8,9], others found no association or reported adverse metabolic effects at high levels of alcohol consumption [10,11]. Moreover, the mechanisms underlying the relationship between alcohol consumption and insulin resistance are not well understood [12,13].

Recent studies have unveiled associations of gut microbiota with insulin resistance and diabetes [14]. Alcohol consumption has also been associated with alterations in the gut microbiota [15]. The mechanism where alcohol consumption alters human gut microbiota is likely complicated [16]. Alcohol is primarily metabolized into acetaldehyde by alcohol dehydrogenase, which is further metabolized by acetaldehyde dehydrogenase into acetate. Upon oral intake, alcohol is rapidly absorbed in the upper part of the small intestine and transported by blood circulation to various organs, including the liver and large bowel. A previous animal study indicated that changes in the gut microbiota related to alcohol consumption may not be directly driven by ethanol but plausibly by acetate [16]. Thus, host genetics might be an important factor in moderating gut microbiota affected by alcohol consumption, as genetic variants have been identified to significantly modify enzymes involved in alcohol metabolism [17,18]. In particular, individuals who carry the *His* variant (*ADH1B* rs1229984 CT/TT genotype) metabolize ethanol to acetaldehyde 70- to 80-fold faster than individuals who do not due to increased enzymatic function, thereby resulting in significantly higher accumulated levels of acetaldehyde in blood [18]. However, to the best of our knowledge, no studies have examined the relationship between alcohol consumption and gut microbiota according to host *ADH1B* genotypes, and how this may relate to insulin resistance is largely unknown.

Therefore, we aimed to identify gut microbial species associated with alcohol consumption in individuals carrying the ADH1B rs1229984 variant and non-carriers from the Hispanic Community Health Study/Study of Latinos (HCHS/SOL). We also examined associations between alcohol consumption and insulin resistance within both genotypes. We then linked the identified alcohol-related gut microbiota with insulin resistance to explore the potential involvement of gut microbiota in the relationship among host genetics, alcohol consumption, and insulin resistance. Based on this framework, we hypothesized that genotype-specific gut microbiota alterations may explain the relationship between alcohol consumption and insulin resistance.

## 2. Methods

### 2.1. Study Design and Population

The HCHS/SOL is an ongoing prospective cohort study of US Hispanics/Latinos, with 16,415 adults aged 18–74 years recruited from four US metropolitan areas (Miami, San Diego, Chicago, and the Bronx area of New York) during the time period 2008–2011 (baseline, V1) [19,20]. Detailed information on sociodemographic, behavior/lifestyle, medication use, and disease history was collected at baseline and updated at the second visit (V2: 2014–2017). In addition, 3035 participants provided fecal samples in a gut microbiome ancillary study (Gut Origins of Latino Diabetes, GOLD) at V2. In the current study, we included 6354 participants who were free of diabetes at V2 and had data on genetics, alcohol consumption, and insulin resistance and related metabolic traits, and a subsample of these participants had gut microbiome data (*n* = 1592). Individuals with diabetes were excluded to minimize potential bias arising from the known effects of diabetes and its treatments (e.g., Metformin use) on gut microbiota composition. Additionally, diabetes may influence alcohol consumption and related lifestyle behaviors, which could confound the associations under investigation. All participants provided written informed consent, and the study was approved by the Institutional Review Boards from each participating institute.

### 2.2. Assessment of Metabolic Traits and Insulin Resistance

Anthropometric and biochemical assessments were performed following standardized methods and protocols [21,22]. BMI was calculated as weight in kilograms divided by height in meters squared. Plasma fasting glucose, 2 h glucose after a standard 75 g oral glucose tolerance test (OGTT), fasting insulin, hemoglobin A1c (HbA1c), high-density lipoprotein cholesterol (HDL-C), low-density lipoprotein cholesterol (LDL-C), and triglycerides were measured using centralized laboratory tests. Diabetes was defined if participants met one of the following criteria as described previously [21]: (1) Fasting glucose ≥ 7.0 mmol/L (126 mg/dL) if fasting time > 8 h or ≥11.1 mmol/L (200 mg/dL) and if fasting time ≤ 8 h; (2) post-2 h glucose ≥ 11.1 mmol/L (200 mg/dL); (3) HbA1c ≥ 6.5%; and (4) current use of antidiabetic medications or self-reported physician-diagnosed T2D. Participants who met the above criteria were excluded from our analysis. HOMA of insulin resistance (HOMA-IR) and HOMA of beta-cell function (HOMA-B) were derived based on: [$\frac{fasting\;glucose\;(mg/dL)*fasting\;insulin\;(mU/L)}{405}$] and 20 × fasting insulin (mU/mL)/[fasting glucose (mg/dL) − 63], respectively [23]. Participants with HOMA-IR ≥ 2.5 were defined as insulin resistant [24,25,26].

### 2.3. Alcohol Consumption Assessment

A questionnaire was administered to determine alcohol consumption. Alcohol use was considered in two ways for analyses. First, a binary-category variable was created, distinguishing between users (currently drinking alcohol) and non-users (including participants who have never consumed alcohol and those who were not presently drinking alcohol but previously drank). Second, an alcohol consumption level variable was generated, delineating participants based on their current weekly alcohol drink level, with 0 for non-users, 1 for those consuming less than the median weekly intake (<2 drinks weekly), 2 for individuals within a weekly intake between the median and the third quartiles (≥2 and <6 drinks weekly), and 3 for those consuming 6 or more drinks weekly.

### 2.4. Genotyping

DNA extracted from blood samples collected from participants at baseline was genotyped using a customized Illumina array (SOL/HCHS Custom 15041502 B3) from Illumina, Inc. (San Diego, CA, USA), which consists of the illumine omni-2.5M array and ~150,000 custom SNPs [27]. Pre-phasing was performed with SHAPEIT2 (v.2.r644), and imputation was performed with IMPUTE2 (v.2.3.0) with the 1000 Genomes Project phase III reference panels. A total of 13,039,987 variants in 22 autosomes among 12,803 samples passed quality filters with MAF (minor alleles frequency) ≥ 1% and imputation quality (Rsq) ≥ 0.3 [27]. Data on SNP rs1229984 at *AHD1B* were analyzed in this study.

### 2.5. Gut Microbiome Profiling

Enrolled GOLD participants were provided with a stool collection kit. For each participant, a single fecal specimen was self-collected using a disposable paper inverted hat (Protocult collection device, ABC Medical Enterprises, Inc., Rochester, MN, USA). Detailed DNA extraction and sequencing procedures were described previously [28,29]. Gut microbiome taxonomic features were defined through the SHOGUN pipeline [30]. In this study, microbiome analyses were conducted for gut microbial species that were present in more than 20% of samples and had an average count greater than 50. Centered log-ratio (CLR) was used to transform abundances of individual gut microbial species.

### 2.6. Statistical Analysis

**Figure 1:**
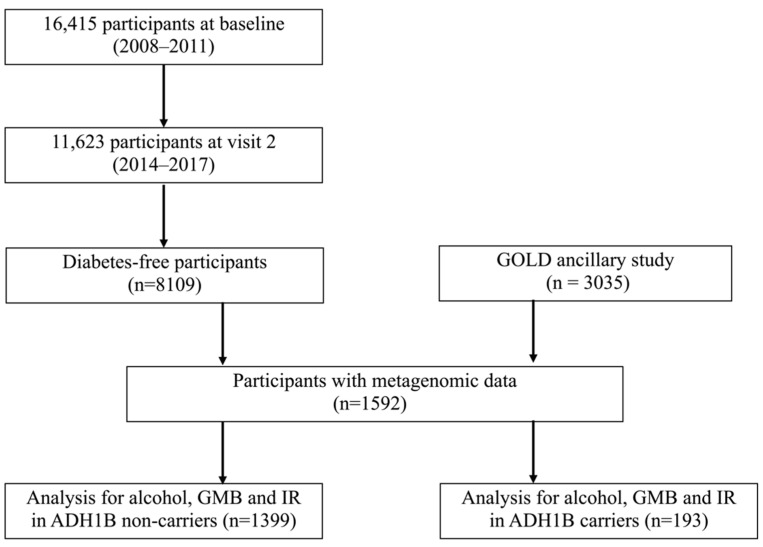
CONSORT diagram showing the flow of participants through the analysis.

A flow chat for participants included in the analyses is shown in Figure 1. Given the known dominant effect of the function variant (*ADH1B* rs1229984) [18] on ethanol metabolism and a few participants who were homozygous, we classified individuals into two genotype groups in the analysis: *ADH1B* rs1229984 T carriers (CT/TT genotype) and non-carriers (CC genotype). For continuous variables and categorical variables, the Wilcoxon rank-based test and χ^2^ test were used for comparison between carriers and non-carriers, respectively. We employed logistic regression to examine the association between alcohol consumption levels and insulin resistance in all participants (*n* = 6354), non-carriers (*n* = 5573), and carriers (*n* = 781), adjusting for age, sex, field center, Hispanic/Latino background, education, annual household income, smoking, US nativity, hypertension, dyslipidemia, and diet quality (measured by alternative Health Eating Index 2010 [AHEI2010]).

Among 1592 participants with gut microbiome data, we employed the function “ancombc()” in R package ANCOMBC (version 2.8.1) to conduct the Analysis of Composition of Microbiomes (ANCOM) [31,32] to identify gut bacterial species associated with alcohol consumption levels in *ADH1B* rs1229984 variant non-carriers (*n* = 1399) and carriers (*n* = 193) separately, adjusting for the covariates mentioned above. Weighted alcohol-associated gut microbiota score (GMS) was calculated based on bacterial species identified in the *AHD1B* variant non-carriers and carriers, respectively. For k identified bacterial species for n participants, the score was calculated as follows:

$$GMS=\beta \Sigma^{-1/2}X,$$

where β is the 1×k vector of the log-fold change of alcohol consumption estimated from ANCOMBC, Σ is the k×k correlation matrix of identified species, and X is the k×n  matrix of CLR-transformed abundances. Levels of GMSs were examined according to alcohol consumption levels by linear regression in carriers and non-carriers separately, adjusting for the covariates mentioned above. Associations of GMSs with insulin resistance were examined by logistic regression in carriers and non-carriers separately, adjusting for the covariates mentioned above.

Among non-carriers, we examined the association between alcohol consumption and insulin resistance stratified by tertiles of the GMS (low, middle, and high), as well as tertiles of individual alcohol-associated bacterial species (CLR-transformed abundances), using logistic regression, adjusting for the covariates mentioned above. Similar analyses were conducted on continuous metabolic traits, including HOMA-IR, 2 h glucose after OGTT, BMI, fasting insulin, fasting glucose, HbA1c, HOMA-B, HDL-C, LDL-C, and triglycerides, using rank-based robust regression models with adjustments for the covariates mentioned above. The alcohol–GMS/species interactions were tested by constructing a product term between the tertiles of GMS/species and alcohol consumption levels in regression models.

R (R project for Statistical Computing, version R-4.2.3) was used for the data manipulation and analysis. False discovery rate (FDR) adjustment was used to account for multiple testing, with a cutoff of 0.05.

## 3. Results

### 3.1. Characteristics of Study Participants

Participant characteristics are shown in Table 1. Among 6354 participants in our analysis, approximately 12% were *ADH1B* variant carriers. The variant allele frequency of ADH1B rs1229984 in this study is comparable to that reported in other Hispanic populations [33,34]. Compared to non-carriers, the carriers were slightly older, had slightly lower BMI, and were less likely to be of Mexican background. As expected, alcohol drinkers comprised less carriers (58%) than non-carriers (62%), and the *ADH1B* genetic variant was negatively associated with alcohol consumption level (*p*-value = 0.002). There was no significant association between the *ADH1B* genetic variant and insulin resistance (*p* = 0.11). Income and education levels did not differ significantly between carriers and non-carriers. Characteristics in a subset of 1592 participants with gut microbiome data were generally similar compared to all participants.

### 3.2. Association Between Alcohol Consumption and Insulin Resistance According to ADH1B Genotype

**Figure 2:**
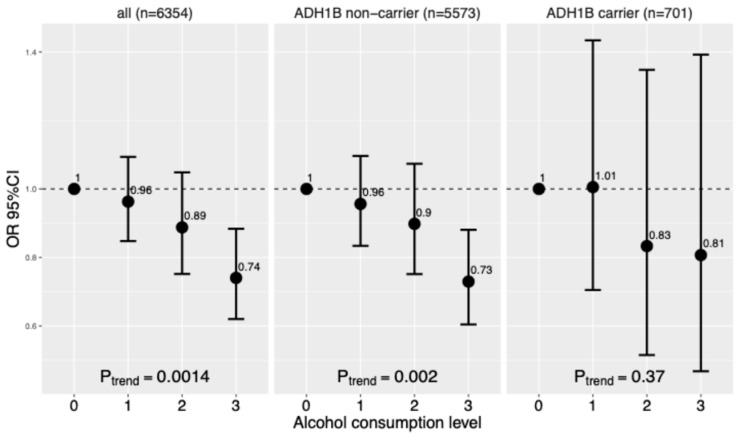
Association between alcohol consumption and insulin resistance (IR) by ADH1B genotypes in 6354 participants who were free of diabetes at Visit 2. Estimates are odds ratios [ORs] and 95% confidence intervals [CIs] for IR estimated by logistic regression, adjusting for age, sex, income, education, study site, US born, smoking, hypertension, dyslipidemia, AHEI2010, income, and Hispanic genetic backgrounds.

We first examined the association between alcohol consumption and insulin resistance among 6354 participants who were free of diabetes. Among all participants, alcohol consumption was negatively associated with insulin resistance (P_trend_ = 0.0014) (Figure 2). Compared to non-drinkers, participants consuming six or more drinks weekly had a lower odds ratio of insulin resistance (OR = 0.74, 95% CI: 0.62–0.88). The association was similar in non-carriers (P_trend_ = 0.002). In carriers, we found a similar trend of association, but it was not significant (P_trend_ = 0.37), which might be due to the relatively small sample size of this group.

### 3.3. Association Between Alcohol Consumption and Gut Microbiota According to ADH1B Genotype

**Figure 3:**
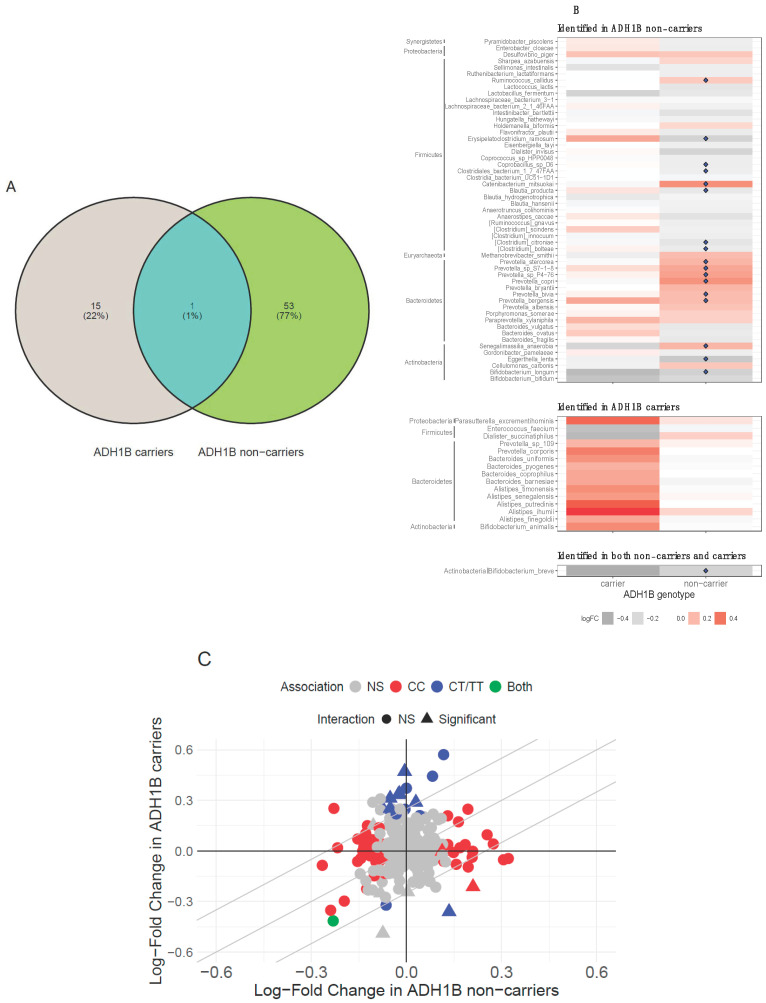
(**A**) Venn diagram of identified species associated with alcohol consumption (*p* < 0.05) in 1399 non-carriers (CC) and 193 carriers (CT/TT). (**B**) Comparison of associations of species identified in both non-carriers, carriers, and both carriers and non-carriers. The color represents log-fold change for association, and ♦ indicates species with FDR-q < 0.05. (**C**) Scatter plot of the log-fold change estimates for alcohol consumption level in carriers and non-carriers. Δ indicates significant ADH1B–alcohol interaction (P_interaction_ < 0.05), which was derived from the test of product term of alcohol consumption level and genotype. Red indicates species significantly associated with alcohol consumption level in non-carriers, blue indicates species significantly associated with alcohol consumption level in carriers, and green indicates both.

Given the important role of the *ADH1B* genotype in the metabolism of alcohol, we examined the associations between alcohol consumption levels and abundances of 229 predominant gut bacterial species (prevalence > 20% and average count > 50) in non-carriers (*n* = 1399) and carriers (*n* = 193), separately. We identified 54 and 16 bacterial species associated with alcohol consumption in non-carriers and carriers (*p* < 0.05), respectively (Figure 3A). In non-carriers, top species positively associated with alcohol consumption level were *Prevotella* (*P.*) species (*P. P4–76*, *P. S7–1–8*, *P. copri*, *P. bergensis*, *P. bivia*, and *P. stercorea*), *Senegalimassilia_anaerobia*, *Catenibacterium_mitsuokai*, and *Ruminococcus_callidus* (*R. callidus*) (FDR-q < 0.05), and top species negatively associated with alcohol consumption level were *Eggerthella_lenta*, *Clostridium_citroniae*, *Bifidobacterium_breve*, *Erysipelatoclostridium_ramosum* (*E. ramosum*), *Bifidobacterium_longum*, *Blautia_producta*, *Coprobacillus_sp._D6*, *Clostridium_bolteae*, and *Clostridiales_bacterium* (FDR-q < 0.05). In carriers, top species positively associated with alcohol consumption were *Bacteroidetes* (such as *Alistipes* spp. and *Bacteroides* spp.) and *Actinobacteria* (such as *Bifidobacterium_animalis* and *Bifidobacterium_breve*), and top species negatively associated with alcohol consumption were *Firmicutes* (such as *Enterococcus_faecium* and *Dialister_succinatiphilus*). However, these species did not remain statistically significant after controlling for multiple testing (FDR-q > 0.05) (Figure 3B).

Overall, the associations of alcohol consumption with gut microbial species appeared different between carriers and non-carriers. Among 54 species identified in non-carriers and 16 species identified in carriers, only one species was identified in both genotypes (Figure 3C). Moreover, significant *ADH1B*–alcohol interactions were identified among 10 of these species (P_interaction_ < 0.05). These results suggested distinct gut microbial profiles associated with alcohol consumption between *ADH1B* variant carriers and non-carriers.

### 3.4. Gut Microbiota Associated with Alcohol Consumption in Relation to Insulin Resistance

**Figure 4:**
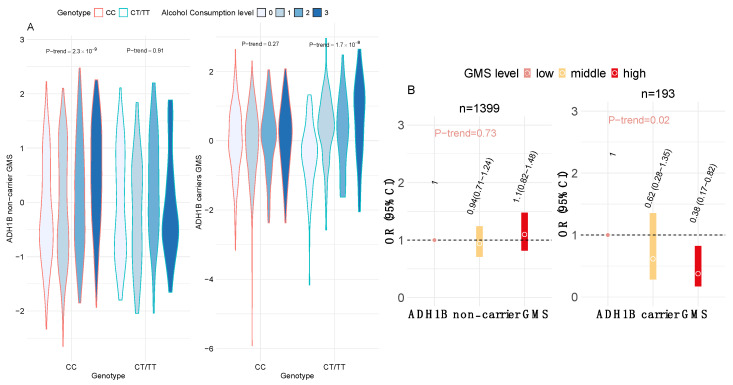
(**A**) Violin plot to compare carrier and non-carrier GMSs and alcohol consumption level in 1399 non-carriers (CC) and 193 carriers (CT/TT). The P_trend_ was obtained by linear regression, in which alcohol consumption level was treated as a continuous variable, adjusting for the covariates mentioned above. (**B**) Association between GMS levels (low, middle, and high) and IR in non-carriers (*n* = 1399) and carriers (*n* = 193). The OR and 95% CI were obtained by logistic regression, adjusting for the covariates mentioned above. The P_trend_ was obtained by treating GMS levels as a continuous variable.

We then calculated the non-carrier GMS and carrier GMS based on 54 and 16 bacterial species associated with alcohol consumption in ADH1B variant non-carriers and carriers, respectively. These GMSs were computed as weighted sums of the relative abundances of alcohol-associated gut microbial species within each genotype group, accounting for inter-species correlation (Methods). Thus, each GMS captures the overall variation in alcohol-associated microbial composition specific to an individual’s ADH1B genotype. We validated the GMSs by assessing their associations with alcohol consumption levels separately in ADH1B carriers and non-carriers. A higher non-carrier GMS was significantly associated with higher alcohol consumption among non-carriers (P_trend_ = 2.3 × 10^−9^) but not among carriers. In contrast, a higher carrier GMS was significantly associated with greater alcohol consumption among carriers (P_trend_ = 1.7 × 10^−8^) but not among non-carriers (Figure 4A). These results support that gut microbial changes associated with alcohol consumption may be specific to the ADH1B genotype. We next examined the associations of these GMSs with insulin resistance. Among carriers, a high carrier GMS was significantly associated with reduced insulin resistance (P_trend_ = 0.02), whereas the non-carrier GMS showed no significant association with insulin resistance (Figure 4B).

### 3.5. Effect Modification of Gut Microbiota on Association Between Alcohol Consumption and Insulin Resistance in Non-Carriers

**Figure 5:**
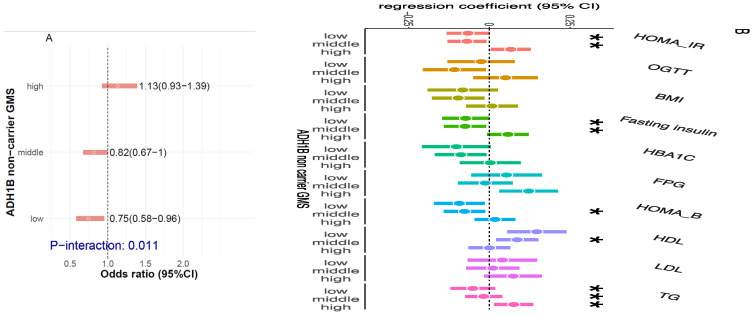
(**A**) The association of alcohol consumption levels with IR by non-carrier GMS levels in non-carriers (*n* = 1399). The participants were classified into three GMS levels (low, middle, and high) by tertiles. In each level, the association of IR with alcohol consumption levels (1, 2, 3, and 4) was assessed by logistic regression, in which alcohol consumption level was treated as a continuous variable, adjusting for the covariates mentioned above. The interaction was assessed by testing the product term of two continuous variables (GMS level and alcohol consumption level) in the logistic regression model. (**B**) The associations of alcohol consumption level with IR-related traits by non-carrier GMS level in non-carriers (*n* = 1399). The participants were classified into three GMS levels (low, middle, and high) by tertiles. In each level, the associations of traits with alcohol consumption levels (1, 2, 3, and 4) were assessed by rank-based robust linear regression, in which alcohol consumption level was treated as a continuous variable, and the covariates mentioned above were adjusted. The interaction was assessed by testing the interaction between two continuous variables (GMS level and alcohol consumption level) in the logistic regression model in the rank-based robust linear regression model. * denotes P_interaction_ < 0.05, ** denotes P_interaction_ < 0.01, and *** denotes P_interaction_ < 0.001.

We then examined whether the identified gut microbiota modified the association between alcohol consumption and insulin resistance. In non-carriers, we found that alcohol consumption was negatively associated with insulin resistance among those with low (1st tertile) and middle (2nd tertile) non-carrier GMSs (OR = 0.75 [95%CI 0.58–0.96] and 0.82 [0.67–1.00], respectively) but not among those with high (3rd tertile) non-carrier GMSs (OR = 1.13 [0.93–1.39]) (P_interaction_ = 0.011) (Figure 5A). Similar findings were observed by comparing drinkers with different alcohol consumption levels to non-drinkers (Supplemental Figure S1). The potential effect modification was also observed for HOMA-IR (P_interaction_ = 0.005), HOMA-B (P_interaction_ = 0.014), fasting insulin (P_interaction_ = 0.006), HDL-C (P_interaction_ = 0.019), and triglycerides (P_interaction_ < 0.001) (Figure 5B). In addition, we also found negative associations of alcohol consumption with BMI, HbA1c, and OGTT among those with low and middle non-carrier GMSs but not in those with high non-carrier GMSs (Figure 5B), though interactions between alcohol consumption and non-carrier GMS were not significant. We did not find such alcohol consumption–GMS interactions on insulin resistance and related metabolic traits in carriers (Supplemental Figure S2A,B).

**Figure 6:**
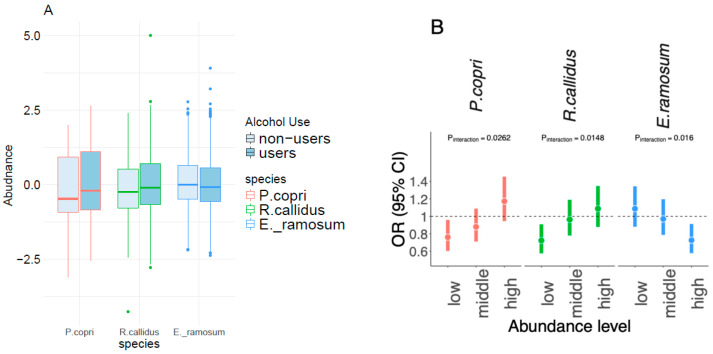
(**A**) The microbiota abundances of alcohol users and non-users for representative species associated with alcohol consumption level (FDR-q < 0.05). (**B**) The modification effects of representative species on the association between alcohol consumption and IR (P_interaction_ < 0.05) in non-carriers (*n* = 1399). The participants were classified into three species abundance levels (low, middle, and high) based on tertiles. In each group, the associations of IR with alcohol consumption levels (1, 2, 3, and 4) were assessed by logistic regression, in which alcohol consumption level was treated as a continuous variable, and covariates mentioned above were adjusted. The differences in associations of groups were assessed by testing the interaction of two continuous variables (abundance tertiles and alcohol consumption level) in the logistic regression model. The colors red, green, and blue represent *P. copri*, *R. callidus*, and *E. ramosum*, respectively.

We next examined whether 18 individual alcohol-related species (FDR-q < 0.05) may modify the association between alcohol consumption level and insulin resistance in non-carriers. We categorized the abundance of bacterial species into three levels (low, middle, and high) based on tertiles. Three species showed significant interactions with alcohol consumption on insulin resistance, including two species that were positively associated with alcohol consumption (*P. copri* and *R. callidus*) and one species that was negatively associated with alcohol consumption (*E. ramosum*) (Figure 6A). For example, alcohol consumption level was negatively associated with insulin resistance in the lowest level of *P. copri*, while it was positively associated with insulin resistance in the highest level (P_interaction_ = 0.026). This trend was similar for *R. callidus* (P_interaction_ = 0.015). In contrast, for *E. ramosum*, alcohol consumption level tended to be positively associated with insulin resistance in its lowest level, while it was negatively associated with insulin resistance in its highest level (P_interaction_ = 0.016) (Figure 6B). Similar results were found for these bacterial species on continuous metabolic traits, especially HOMA-IR and fasting insulin (Supplemental Figure S3).

## 4. Discussion

**Figure 7:**
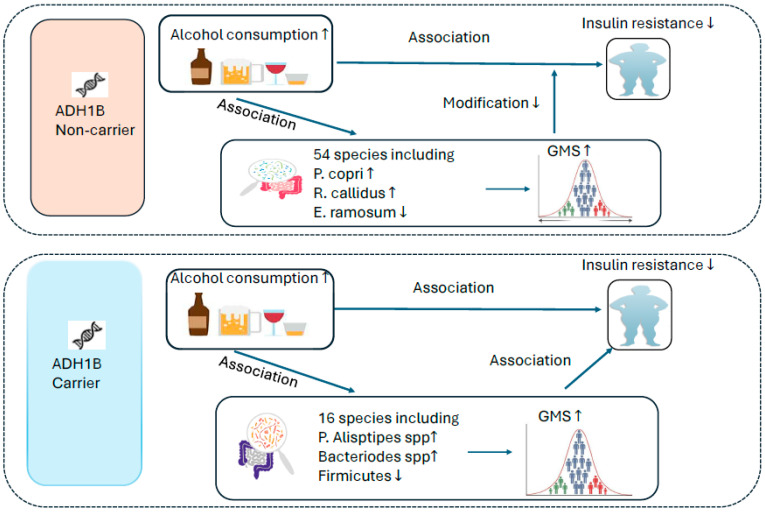
Diagram figure illustrating the findings of this study.

The findings of this study are illustrated in Figure 7. In summary, we identified distinct gut microbial profiles associated with alcohol consumption between the *ADH1B* variant carriers and non-carriers in US Hispanic/Latino adults. Specifically, there were 16 and 54 bacterial species associated with alcohol consumption in carriers and non-carriers, respectively, and only 1 species was associated with alcohol consumption in both groups. We also found that a gut microbiota score based on 16 carrier-specific alcohol-related species showed a negative association with insulin resistance in carriers. In non-carriers, a gut microbiota score based on 54 non-carrier-specific alcohol-related species, as well as several individual species (e.g., *P. copri*), modified the association between alcohol consumption and insulin resistance, with a protective association observed in individuals with a low gut microbiota score or low abundance of *P. copri* (Figure 7). These findings advance our understanding of the interplays between *ADH1B* genotype, alcohol consumption, and gut microbiota in relation to insulin resistance.

One major finding from our study is that gut microbiota associated with alcohol consumption varied across *ADH1B* (rs1229984) genotypes. Individuals who carry the *His* allele of rs1229984 metabolize ethanol to acetaldehyde dramatically faster than non-carriers [18], leading to significantly increased blood acetaldehyde levels after alcohol consumption and, consequently, increased acetaldehyde levels in the intestines. Gut microbiota responding to varied acetaldehyde levels might account for the different alcohol-related gut bacterial species observed in the *ADH1B* variant carriers and non-carriers. Acetaldehyde is considered a major toxic metabolite responsible for various alcohol-related diseases, such as alcoholic liver diseases and cancers [35]. Hence, gut microbial species metabolizing acetaldehyde could be protective against elevated acetaldehyde levels after alcohol consumption in carriers. Our study suggested that species including *Alistipes* spp. and *Bacteroides* spp. might be increased by alcohol consumption in carriers. Previous studies have suggested that *Bacteriodes* might have potential for treating ethanol-induced liver damage [36,37]. Meanwhile, *Alistipes* has also been implicated in liver disease [38] and colorectal cancer [39]. However, it is unclear if these alcohol-associated species in the *ADH1B* carriers are causal or side-products of alcohol consumption. Future studies are needed to disentangle the effects of alcohol consumption on gut microbiota and the effects of the alcohol-induced microbiome alterations on these diseases.

Another interesting finding of our study is that gut microbiota associated with alcohol consumption in the *ADH1B* non-carriers might modify the relationship between alcohol consumption and insulin resistance. We constructed a microbiome score based on alcohol-associated microbiota in the *ADH1B* non-carriers and found that higher levels of alcohol consumption were associated with lower odds of insulin resistance only in individuals with lower microbiome scores but not those with higher microbiome scores. This suggested that the potential beneficial effects of alcohol consumption on insulin resistance may be weakened by altered gut microbiota. Epidemiological studies have investigated the relationship between alcohol consumption and insulin resistance, as well as T2D, but the results have been highly heterogeneous [11,40]. The identified microbiome–alcohol interaction on insulin resistance may partially explain this heterogeneity. Furthermore, our individual species analysis identified several gut bacterial species which might modify the association between alcohol consumption and insulin resistance in the *ADH1B* non-carriers. One of the most interesting identified species was *P. copri*, which was positively associated with alcohol consumption, while the favorable association between alcohol consumption and insulin resistance was only observed in individuals with a low abundance of this species. *P. copri*, the most abundant species of *Prevotella* in the human gut, has been positively associated with healthy plant-rich/fiber-rich diets, but its impacts on human health are contradictory [41]. For example, positive associations of *P. copri* with obesity [42] and insulin resistance [43] were reported in previous studies. Several hypotheses have been developed behind the conflicting observations on its association with diet and human health, such as strain-level diversity [44,45], bacterial co-occurrence [29], and epiphenomena [46]. Our results suggested another possibility: that the diminished beneficial effects of alcohol consumption upon the increased abundance of *P. copri* may be due to the reduction of ethanol-derived functional metabolites, such as acetate, which has been implicated in weight loss and improved insulin sensitivity [47,48]. It has been shown that gut microbiota responds to ethanol feeding by activating acetate dissimilation, not by metabolizing ethanol directly [16]. Although it is unknown whether *Prevotella species* can use acetate directly, a previous mouse study demonstrated that *Prevotella species* may alter the composition and function of the ecosystem, resulting in a reduction of acetate [49]. Notably, acetate can also come from healthy dietary sources, particularly high-fiber diets [50]. In line with our hypothesis, previous studies also reported that the beneficial associations of the Mediterranean diet, a healthy plant-rich/fiber-rich diet, with diabetes and cardiometabolic disease risk were only observed among individuals with a decreased abundance of *P. copri* [51,52].

On the other hand, our analyses suggested that the carrier GMS score tended to be favorably associated with insulin resistance. However, due to a small number of carriers in our analysis, future studies are needed to validate such protective effects of gut microbiota on insulin resistance. Taken together, our findings suggested that the association between alcohol consumption and insulin resistance could be complicated, as it may be modified by gut microbiota. Hence, recommendations to consume alcohol for metabolic health should be made with caution, especially considering the link between alcohol consumption and other well-documented detrimental health effects.

This study has several limitations. The population-based cross-sectional data in assessments of the relationship between gut microbiota and insulin resistance might be biased by reverse association. Additionally, due to the observational nature of the data, this epidemiological study may also be biased by numerous confounding factors. Although our analysis controlled for multiple potential confounders, residual and other unknown confounding effects may still exist. Hence, our analysis was unable to make causal inference, and future investigations including experimental studies are needed to validate our findings. Furthermore, due to the distribution of *ADH1B* genotypes in Hispanic/Latino populations, our analyses included a relatively small sample size of *ADH1B* variant carriers, which limited our statistical power to detect gut bacterial species associated with alcohol consumption specifically in this group. Finally, the study population comprised US Hispanics/Latinos with diverse backgrounds. Although we performed shotgun metagenomic sequencing, the sequencing depth in our study was insufficient to support sub-species or strain-level analysis of the microbiota. Previously, significant variation in the microbiome composition of different populations through environmental, dietary, and host genetic factors was reported [53]. For example, *P. copri* displays significant functional diversity across clades, which are geographically distributed, not only at the clade level but also at the intra-clade level [54]. Studies in ethnogeographically diverse populations using deeper sequencing approaches could further assess the generalizability of our finding regarding the role of *P. copri* in the relationship between alcohol consumption and insulin resistance.

## 5. Conclusions

Our study suggested a gene–alcohol interaction on gut microbiota and potentially different roles of alcohol-related gut microbiota in the links between alcohol consumption and insulin resistance between the *ADH1B* variant carriers and non-carriers. Although this study has limitations in inferring causal relationships, it provides valuable insights into potential gut microbiota-related mechanisms underlying the relationship between alcohol consumption and insulin resistance. These insights can potentially lead to the development of strategies for reshaping the gut microbiome to improve personalized metabolic health.

## Figures and Tables

**Table 1:** Characteristics of participants.

	All Subjects (6354)			Subjects with Microbiome Data (1592)		
	Carrier	Non-Carrier	*p*-Value	Carrier	Non-Carrier	*p*-Value
n (%)	781 (12)	5573 (88)		193 (12)	1399 (88)	
Age (Years)	53 (43–62)	52 (41–60)	0.01	56 (51–63)	56 (51–63)	0.97
Male (%)	307 (39)	2061 (37)	0.22	79 (41)	477 (34)	0.074
US Born (%)	146 (19)	985 (18)	0.52	45 (23)	220 (16)	0.011
Current Smoker (%)	135 (17)	886 (16)	0.54	41 (21)	192 (14)	0.008
Income (%)						0.93
Less than USD 30,000	407 (52)	2897 (52)	0.46	107 (55)	792 (57)	
USD 30,000 or more	331 (42)	2305 (41)		78 (40)	555 (40)	
Missing	43(6)	371 (7)		8 (4)	52 (4)	
Education			0.38			0.52
Less than High School	252 (32)	1845 (33)		68 (35)	478 (34)	
High School or Equivalent	194 (25)	1478 (27)		39 (20)	335 (24)	
Greater than High School or Equivalent	334 (43)	2244 (40)		85 (44)	581 (42)	
Genetic Backgrounds			0.01			0.67
Central American	88 (11)	627 (11)		19 (10)	133 (10)	
Cuban	174 (22)	952 (17)		40 (16)	228 (16)	
Dominican	72 (9)	508 (9)		17 (9)	152 (11)	
Mexican	267 (34)	2171(39)		67 (35)	524 (38)	
Puerto Rican	108 (14)	832 (15)		33 (17)	246 (18)	
South American	72 (9)	468 (8)		17 (9)	112 (8)	
Alcohol Consumption (%)			0.002 *			0.77
Non-drinker	331 (42)	2128 (38)		86 (45)	564 (40)	
<2 drinks a week	258 (33)	1824 (33)		54 (28)	477 (34)	
>2 and <6 drinks a week	109 (14)	820 (15)		30 (16)	193 (14)	
>6 drinks a week	83 (11)	792 (14)		23 (12)	165 (12)	
Insulin Resistance (%)	425 (55)	3200 (58)	0.11	109 (57)	801 (58)	0.90
BMI (kg/m^2^)	28.14 (25.37–31.87)	28.82 (25.84–32.62)	0.004	28.56 (25.34–32.30)	29.04 (26.01–32.36)	0.40
Fasting glucose (mg/dL)	95 (90–101)	96 (90–102)	0.33	94 (89–99)	93 (89–99)	0.89
HbA1c (mmol mol^−1^)	38 (34–40)	38 (36–40)	0.11	38 (36–40)	38 (36–41)	0.21
Fasting insulin (mIU/L)	11.33 (7.83–17.00)	11.83 (8.00–17.67)	0.17	11.50 (5.50–9.00)	12 (7.83–18.54)	0.84
HDL-C (mg/dL)	50 (42–61)	50 (41–60)	0.53	49 (42–59)	51 (42–62)	0.21
LDL-C (mg/dL)	116 (95–137)	116 (96–139)	0.57	118 (97.75 (140.25)	120 (98–142)	0.53
Triglycerides (mg/dL)	100 (69–139)	101 (71–146)	0.21	100.50 (68–135)	100 (73–145)	0.47
2 h glucose after OGTT (mg/dL)	116 (95–139)	116 (98–140)	0.24	112 (94–143)	119 (100–144)	0.03
HOMA-B	130.91 (90.00–182.77)	131.39 (92.14–192.00)	0.48	131.01 (86.67–190.29)	130 (90.72–182.69)	0.71
HOMA-IR	2.69 (1.89–4.16)	2.89 (1.85–4.32)	0.14	2.84 (1.87–4.52)	2.81 (1.85–4.15)	0.87

Values are denoted as median (IQR1, IQR3). Continuous variables were tested using Wilcox rank-based test. Categorical variables were tested using chi-square test. *: *p*-value is obtained by a logistic regression for testing the trend between variant carrier and alcohol consumption level.

## Data Availability

The genetics data of HCHS/SOL used in this paper are archived at the National Institutes of Health repositories dbGap (accession number phs000810.v1.p1) and BIOLINCC (accession number HLB01141418a), and gut microbiome sequence data in this study are deposited in QIITA (ID 11666) and EMBL-EBI ENA (ERP117287). HCHS/SOL has established a process for the scientific community to apply for access to participant data and materials, with such requests reviewed by the project’s Steering Committee. These policies are described at https://sites.cscc.unc.edu/hchs/ (accessed on 14 August 2025). The corresponding author will accept reasonable requests for data access, which will be referred to the Steering Committee of the HCHS/SOL project. No custom codes or functions were generated in this study. All codes regarding the main analyses or data visualization can be available upon reasonable request to the corresponding author.

## Supplementary Materials

The following supporting information can be downloaded at: https://www.mdpi.com/article/10.3390/nu17162669/s1, Figure S1: The association of alcohol consumption levels with IR by non-carrier GMS levels in non-carriers (*n* = 1399). The participants were classified into three GMS levels (low, middle, and high) by tertiles. In each level, the association of IR with alcohol consumption levels (non-drinker, <median, <75%, >75%, respectively) were assessed by logistic regression, in which non-drinkers were treated as the reference group, adjusting for covariates mentioned above. Figure S2: (**A**) The association of alcohol consumption levels with IR by carrier GMS levels in carriers (*n* = 193). The participants were classified into three GMS levels (low, middle, and high) by tertiles. In each level, the association of IR with alcohol consumption levels (1–4) was assessed by logistic regression, in which alcohol consumption level was treated as a continuous variable, adjusting for covariates mentioned above. The interaction was assessed by testing the product terms of two continuous variables (GMS level and alcohol consumption level) in the logistic regression model. (**B**) The associations of alcohol consumption level with IR-related traits by carrier GMS level in carriers (*n* = 193). The participants were classified into three GMS levels (low, middle, and high) by tertiles. In each level, the associations of traits with alcohol consumption levels (1–4) were assessed by rank-based robust linear regression in which alcohol consumption level was treated as a continuous variable and covariates mentioned above were adjusted. The interaction was assessed by testing the interaction between two continuous variables (GMS level and alcohol consumption level) in the logistic regression model in the rank-based robust linear regression model. Figure S3: The modification effects of representative species on the association between alcohol consumption and glycemic traits among representative species abundance levels in non-carriers (*n* = 1399). The participants were classified into three species abundance levels (low, middle, and high) by tertiles. In each group, the associations of glycemic traits with alcohol consumption levels (1–4) were assessed by rank-based robust linear regression in which alcohol consumption level was treated as a continuous variable and covariates mentioned above were adjusted. The differences in associations of groups were assessed by testing the interaction between two continuous variables (abundance tertiles and alcohol consumption level) in the robust regression model. * denotes P_interaction_ < 0.05, and ** denotes P_interaction_ < 0.01.

## References

[1] Despres J.P., Lemieux I. (2006). Abdominal obesity and metabolic syndrome. Nature.

[2] Wang L., Li X., Wang Z., Bancks M.P., Carnethon M.R., Greenland P., Feng Y.-Q., Wang H., Zhong V.W. (2021). Trends in prevalence of diabetes and control of risk factors in diabetes among US adults, 1999–2018. JAMA.

[3] Chatterjee S., Khunti K., Davies M.J. (2017). Type 2 diabetes. Lancet.

[4] Laakso M., Kuusisto J. (2014). Insulin resistance and hyperglycaemia in cardiovascular disease development. Nat. Rev. Endocrinol..

[5] Rydén L., Grant P.J., Anker S.D., Berne C., Cosentino F., Danchin N., Deaton C., Escaned J., Task Force on diabetes, pre-diabetes, and cardiovascular diseases of the European Society of Cardiology (ESC), European Association for the Study of Diabetes (EASD) (2014). ESC guidelines on diabetes, pre-diabetes, and cardiovascular diseases developed in collaboration with the EASD—Summary. Diab. Vasc. Dis. Res..

[6] Brown A.E., Walker M. (2016). Genetics of Insulin Resistance and the Metabolic Syndrome. Curr. Cardiol. Rep..

[7] Shigeta H., Shigeta M., Nakazawa A., Nakamura N., Yoshikawa T. (2001). Lifestyle, obesity, and insulin resistance. Diabetes Care.

[8] Freiberg M.S., Cabral H.J., Heeren T.C., Vasan R.S., Ellison R.C., Third National Health and Nutrition Examination Survey (2004). Alcohol consumption and the prevalence of the Metabolic Syndrome in the US.: A cross-sectional analysis of data from the Third National Health and Nutrition Examination Survey. Diabetes Care.

[9] Hendriks H.F.J. (2007). Moderate alcohol consumption and insulin sensitivity: Observations and possible mechanisms. Ann. Epidemiol..

[10] Cordain L., Melby C.L., E Hamamoto A., O’NEill D., Cornier M.-A., A Barakat H., Israel R.G., O Hill J. (2000). Influence of moderate chronic wine consumption on insulin sensitivity and other correlates of syndrome X in moderately obese women. Metabolism.

[11] Schrieks I.C., Heil A.L., Hendriks H.F., Mukamal K.J., Beulens J.W. (2015). The effect of alcohol consumption on insulin sensitivity and glycemic status: A systematic review and meta-analysis of intervention studies. Diabetes Care.

[12] Lazarus R., Sparrow D., Weiss S.T. (1997). Alcohol intake and insulin levels. The Normative Aging Study. Am. J. Epidemiol..

[13] Kiechl S., Willeit J., Poewe W., Egger G., Oberhollenzer F., Muggeo M., Bonora E. (1996). Insulin sensitivity and regular alcohol consumption: Large, prospective, cross sectional population study (Bruneck study). BMJ.

[14] Gou W., Ling C.-W., He Y., Jiang Z., Fu Y., Xu F., Miao Z., Sun T.-Y., Lin J.-S., Zhu H.-L. (2021). Interpretable Machine Learning Framework Reveals Robust Gut Microbiome Features Associated with Type 2 Diabetes. Diabetes Care.

[15] Mutlu E.A., Gillevet P.M., Rangwala H., Sikaroodi M., Naqvi A., Engen P.A., Kwasny M., Lau C.K., Keshavarzian A. (2012). Colonic microbiome is altered in alcoholism. Am. J. Physiol. Gastrointest. Liver Physiol..

[16] Martino C., Zaramela L.S., Gao B., Embree M., Tarasova J., Parker S.J., Wang Y., Chu H., Chen P., Lee K.-C. (2022). Acetate reprograms gut microbiota during alcohol consumption. Nat. Commun..

[17] Wall T.L., Luczak S.E., Hiller-Sturmhofel S. (2016). Biology, Genetics, and Environment: Underlying Factors Influencing Alcohol Metabolism. Alcohol. Res..

[18] Edenberg H.J. (2007). The genetics of alcohol metabolism: Role of alcohol dehydrogenase and aldehyde dehydrogenase variants. Alcohol. Res. Health.

[19] LaVange L.M., Kalsbeek W.D., Sorlie P.D., Avilés-Santa L.M., Kaplan R.C., Barnhart J., Liu K., Giachello A., Lee D.J., Ryan J. (2010). Sample design and cohort selection in the Hispanic Community Health Study/Study of Latinos. Ann. Epidemiol..

[20] Sorlie P.D., Aviles-Santa M.L., Wassertheil-Smoller S., Kaplan R.C., Daviglus M.L., Giachello A.L., Schneiderman N., Raij L., Talavera G., Allison M. (2010). Design and implementation of the Hispanic Community Health Study/Study of Latinos. Ann. Epidemiol..

[21] Qi Q., Li J., Yu B., Moon J.-Y., Chai J.C., Merino J., Hu J., Ruiz-Canela M., Rebholz C., Wang Z. (2022). Host and gut microbial tryptophan metabolism and type 2 diabetes: An integrative analysis of host genetics, diet, gut microbiome and circulating metabolites in cohort studies. Gut.

[22] Qi Q., Strizich G., Merchant G., Sotres-Alvarez D., Buelna C., Castañeda S.F., Gallo L.C., Cai J., Gellman M.D., Isasi C.R. (2015). Objectively Measured Sedentary Time and Cardiometabolic Biomarkers in US Hispanic/Latino Adults: The Hispanic Community Health Study/Study of Latinos (HCHS/SOL). Circulation.

[23] Matthews D.R., Hosker J.P., Rudenski A.S., Naylor B.A., Treacher D.F., Turner R.C. (1985). Homeostasis model assessment: Insulin resistance and beta-cell function from fasting plasma glucose and insulin concentrations in man. Diabetologia.

[24] Reaven G.M. (2003). The insulin resistance syndrome. Curr. Atheroscler. Rep..

[25] Nakai Y., Fukushima M., Nakaishi S., Kishimoto H., Seino Y., Nagasaka S., Sakai M., Taniguchi A. (2002). The threshold value for insulin resistance on homeostasis model assessment of insulin sensitivity. Diabet. Med..

[26] Takeuchi T., Kubota T., Nakanishi Y., Tsugawa H., Suda W., Kwon A.T.-J., Yazaki J., Ikeda K., Nemoto S., Mochizuki Y. (2023). Gut microbial carbohydrate metabolism contributes to insulin resistance. Nature.

[27] Conomos M.P., Laurie C.A., Stilp A.M., Gogarten S.M., McHugh C.P., Nelson S.C., Sofer T., Fernández-Rhodes L., Justice A.E., Graff M. (2016). Genetic Diversity and Association Studies in US Hispanic/Latino Populations: Applications in the Hispanic Community Health Study/Study of Latinos. Am. J. Hum. Genet..

[28] Kaplan R.C., Wang Z., Usyk M., Sotres-Alvarez D., Daviglus M.L., Schneiderman N., Talavera G.A., Gellman M.D., Thyagarajan B., Moon J.-Y. (2019). Gut microbiome composition in the Hispanic Community Health Study/Study of Latinos is shaped by geographic relocation, environmental factors, and obesity. Genome Biol..

[29] Wang Z., Usyk M., Vázquez-Baeza Y., Chen G.-C., Isasi C.R., Williams-Nguyen J.S., Hua S., McDonald D., Thyagarajan B., Daviglus M.L. (2021). Microbial co-occurrence complicates associations of gut microbiome with US immigration, dietary intake and obesity. Genome Biol..

[30] Hillmann B., Al-Ghalith G.A., Shields-Cutler R.R., Zhu Q., Knight R., Knights D. (2020). SHOGUN: A modular, accurate and scalable framework for microbiome quantification. Bioinformatics.

[31] Lin H., Peddada S.D. (2020). Analysis of compositions of microbiomes with bias correction. Nat. Commun..

[32] Lin H., Eggesbo M., Peddada S.D. (2022). Linear and nonlinear correlation estimators unveil undescribed taxa interactions in microbiome data. Nat. Commun..

[33] Ehlers C.L., Liang T., Gizer I.R. (2012). ADH and ALDH polymorphisms and alcohol dependence in Mexican and Native Americans. Am. J. Drug Alcohol Abuse.

[34] Vilar-Gomez E., Sookoian S., Pirola C.J., Liang T., Gawrieh S., Cummings O., Liu W., Chalasani N.P. (2020). ADH1B∗2 Is Associated With Reduced Severity of Nonalcoholic Fatty Liver Disease in Adults, Independent of Alcohol Consumption. Gastroenterology.

[35] Setshedi M., Wands J.R., Monte S.M. (2010). Acetaldehyde adducts in alcoholic liver disease. Oxid. Med. Cell. Longev..

[36] Sangineto M., Grander C., Grabherr F., Mayr L., Enrich B., Schwärzler J., Dallio M., Bukke V.N., Moola A., Moschetta A. (2022). Recovery of Bacteroides thetaiotaomicron ameliorates hepatic steatosis in experimental alcohol-related liver disease. Gut Microbes.

[37] Wang H., Wang Q., Yang C., Guo M., Cui X., Jing Z., Liu Y., Qiao W., Qi H., Zhang H. (2022). Bacteroides acidifaciens in the gut plays a protective role against CD95-mediated liver injury. Gut Microbes.

[38] Rau M., Rehman A., Dittrich M., Groen A.K., Hermanns H.M., Seyfried F., Beyersdorf N., Dandekar T., Rosenstiel P., Geier A. (2018). Fecal SCFAs and SCFA-producing bacteria in gut microbiome of human NAFLD as a putative link to systemic T-cell activation and advanced disease. United Eur. Gastroenterol. J..

[39] Moschen A.R., Gerner R.R., Wang J., Klepsch V., Adolph T.E., Reider S.J., Hackl H., Pfister A., Schilling J., Moser P.L. (2016). Lipocalin 2 Protects from Inflammation and Tumorigenesis Associated with Gut Microbiota Alterations. Cell Host Microbe.

[40] Joosten M.M., Chiuve S.E., Mukamal K.J., Hu F.B., Hendriks H.F., Rimm E.B. (2011). Changes in alcohol consumption and subsequent risk of type 2 diabetes in men. Diabetes.

[41] Abdelsalam N.A., Hegazy S.M., Aziz R.K. (2023). The curious case of *Prevotella copri*. Gut Microbes.

[42] Thingholm L.B., Rühlemann M.C., Koch M., Fuqua B., Laucke G., Boehm R., Bang C., Franzosa E.A., Hübenthal M., Rahnavard A. (2019). Obese Individuals with and without Type 2 Diabetes Show Different Gut Microbial Functional Capacity and Composition. Cell Host Microbe.

[43] Pedersen H.K., Gudmundsdottir V., Nielsen H.B., Hyotylainen T., Nielsen T., Jensen B.A.H., Forslund K., Hildebrand F., Prifti E., Falony G. (2016). Human gut microbes impact host serum metabolome and insulin sensitivity. Nature.

[44] Ley R.E. (2016). Gut microbiota in 2015: Prevotella in the gut: Choose carefully. Nat. Rev. Gastroenterol. Hepatol..

[45] De Filippis F., Pasolli E., Tett A., Tarallo S., Naccarati A., De Angelis M., Neviani E., Cocolin L., Gobbetti M., Segata N. (2019). Distinct Genetic and Functional Traits of Human Intestinal Prevotella copri Strains Are Associated with Different Habitual Diets. Cell Host Microbe.

[46] Janket S.J., Conte H.A., Diamandis E.P. (2021). Do Prevotella copri and Blastocystis promote euglycaemia?. Lancet Microbe.

[47] Hernandez M.A.G., Canfora E.E., Jocken J.W.E., Blaak E.E. (2019). The Short-Chain Fatty Acid Acetate in Body Weight Control and Insulin Sensitivity. Nutrients.

[48] Moffett J.R., Puthillathu N., Vengilote R., Jaworski D.M., Namboodiri A.M. (2020). Acetate Revisited: A Key Biomolecule at the Nexus of Metabolism, Epigenetics and Oncogenesis-Part 1: Acetyl-CoA, Acetogenesis and Acyl-CoA Short-Chain Synthetases. Front. Physiol..

[49] Iljazovic A., Roy U., Gálvez E.J.C., Lesker T.R., Zhao B., Gronow A., Amend L., Will S.E., Hofmann J.D., Pils M.C. (2021). Perturbation of the gut microbiome by Prevotella spp. enhances host susceptibility to mucosal inflammation. Mucosal Immunol..

[50] Mueller N.T., Zhang M., Juraschek S.P., Miller E.R., Appel L.J. (2020). Effects of high-fiber diets enriched with carbohydrate, protein, or unsaturated fat on circulating short chain fatty acids: Results from the OmniHeart randomized trial. Am. J. Clin. Nutr..

[51] Wang D.D., Nguyen L.H., Li Y., Yan Y., Ma W., Rinott E., Ivey K.L., Shai I., Willett W.C., Hu F.B. (2021). The gut microbiome modulates the protective association between a Mediterranean diet and cardiometabolic disease risk. Nat. Med..

[52] Wang D.D., Qi Q., Wang Z., Usyk M., Sotres-Alvarez D., Mattei J., Tamez M., Gellman M.D., Daviglus M., Hu F.B. (2022). The Gut Microbiome Modifies the Association Between a Mediterranean Diet and Diabetes in USA Hispanic/Latino Population. J. Clin. Endocrinol. Metab..

[53] Yatsunenko T., Rey F.E., Manary M.J., Trehan I., Dominguez-Bello M.G., Contreras M., Magris M., Hidalgo G., Baldassano R.N., Anokhin A.P. (2012). Human gut microbiome viewed across age and geography. Nature.

[54] Tett A., Huang K.D., Asnicar F., Fehlner-Peach H., Pasolli E., Karcher N., Armanini F., Manghi P., Bonham K., Zolfo M. (2019). The Prevotella copri Complex Comprises Four Distinct Clades Underrepresented in Westernized Populations. Cell Host Microbe.

