# Supplementary material for: Interplays of ADH1B Genotype, Alcohol Consumption, and Gut Microbiota in Relation to Insulin Resistance
Source: Nutrients. 2025 Aug 18;17(16):2669. doi: 10.3390/nu17162669 (PMC12389023; doi:10.3390/nu17162669)
Supplement: Supplementary file 1 [file nutrients-17-02669-s001.zip › nutrients-3733788-supplementary.pdf]

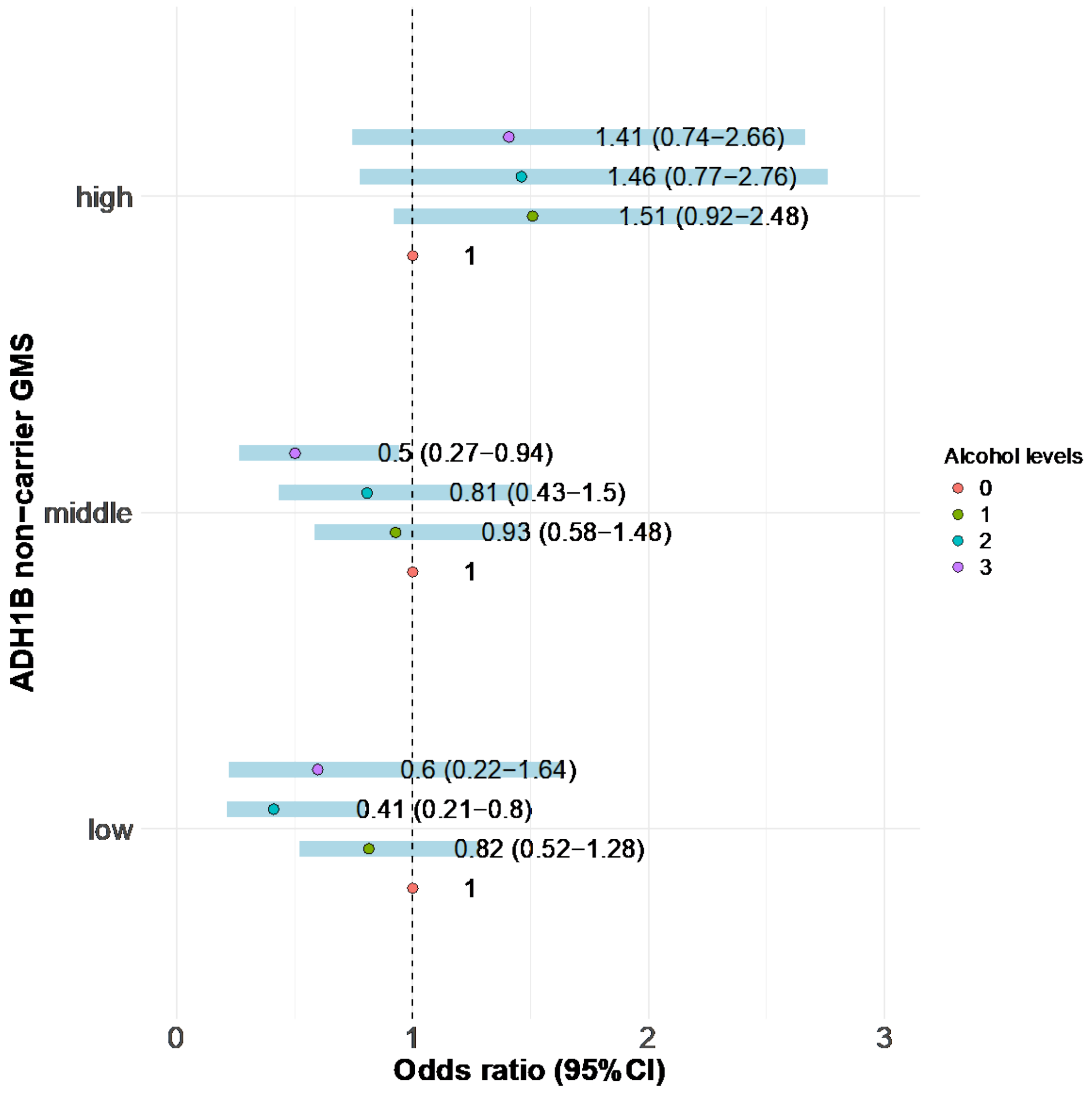

Figure S1 The association of alcohol consumption levels with IR by non-carrier GMS levels in non-carriers (n=1,399). The participants were classified into three GMS levels (low, middle and high) by tertiles. In each level, the association of IR with alcohol consumption levels (0, 1, 2, and 3, respectively) were assessed by logistic regression in which nondrinkers were treated as the reference group, adjusting for covariates mentioned above.

A

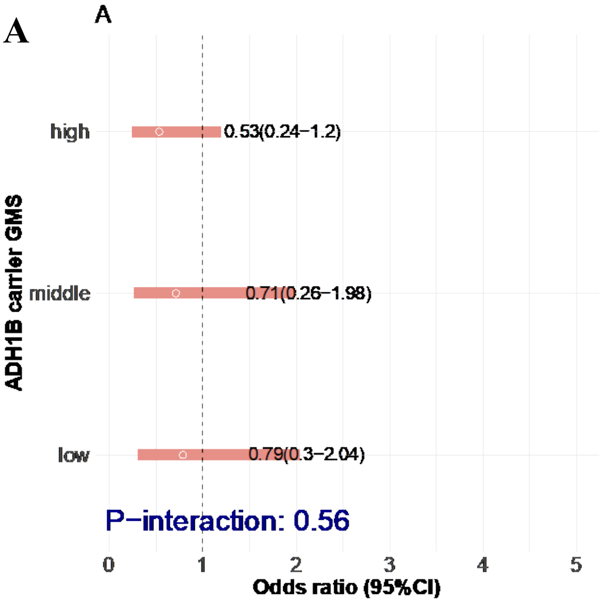

B

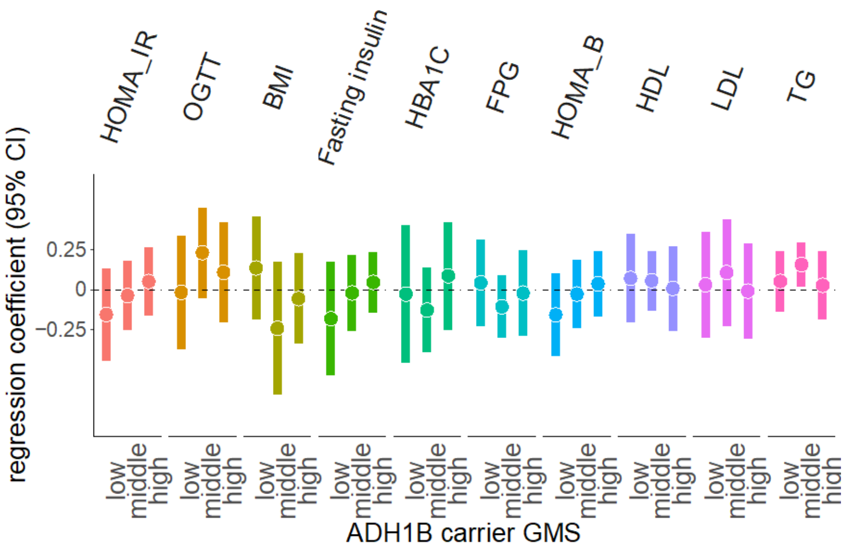

Figure S2 (A) The association of alcohol consumption levels with IR by carrier GMS levels in carriers (n=193). The participants were classified into three GMS levels (low, middle and high) by tertiles. In each level, the association of IR with alcohol consumption levels (1,2,3,4) was assessed by logistic regression in which alcohol consumption level was treated as a continuous variable, adjusting for covariates mentioned above. The interaction was assessed by testing the product term of two continuous variable (GMS level and alcohol consumption level) in the logistic regression model. **(B)** The associations of alcohol consumption level with IR-related traits by carrier GMS level in carriers (n=193). The participants were classified into three GMS level (low, middle and high) by tertiles. In each level, the associations of traits with alcohol consumption levels (0,1,2, 3) were assessed by rank-based robust linear regression in which alcohol consumption level was treated as a continuous variable and covariates mentioned above were adjusted. The interaction was assessed by testing the interaction between two continucomsous variable (GMS level and alcohol consumption level) in the logistic regression model in the rank-based robust linear regression model.

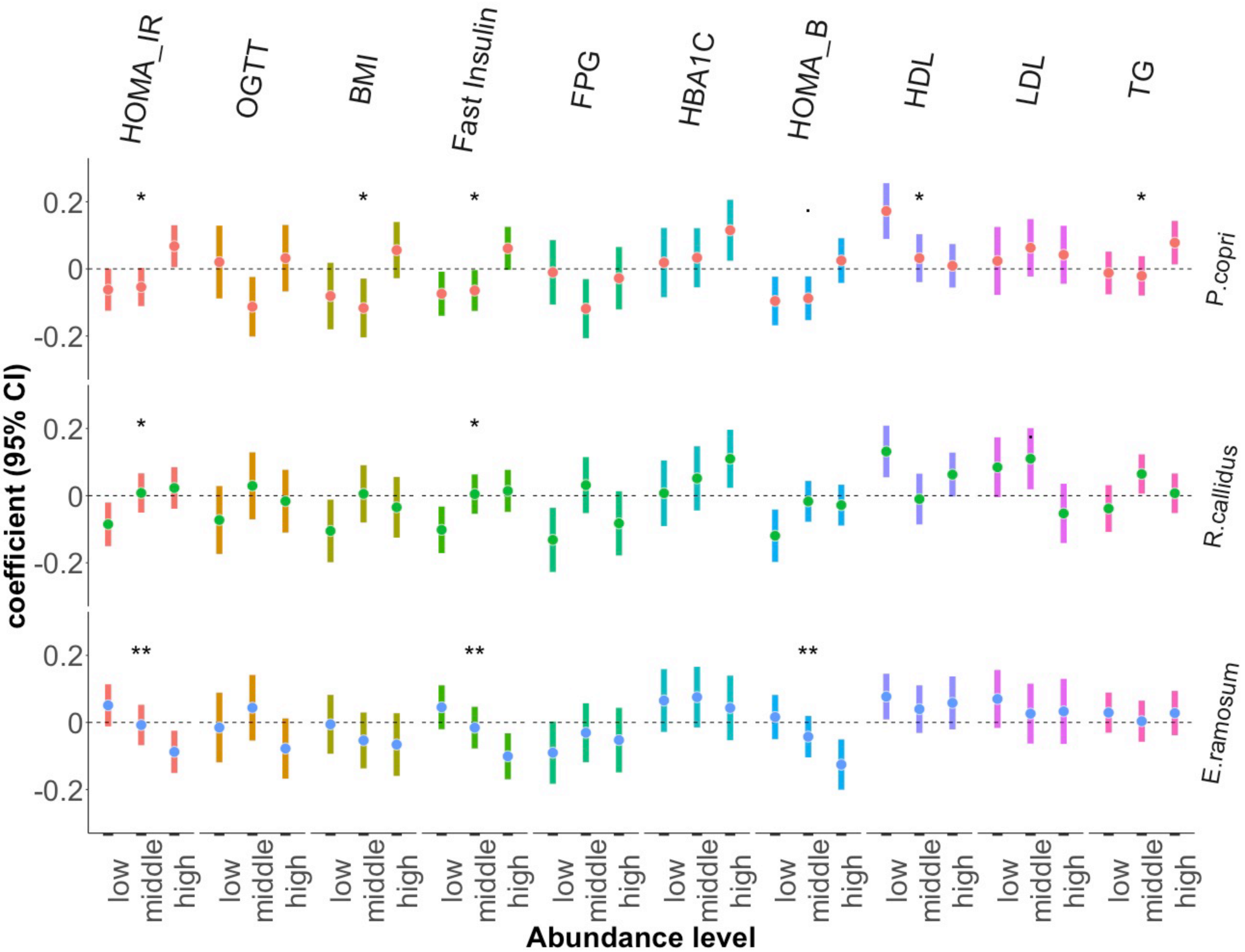

Figure S3 The modification effects of representative species on the association between alcohol consumption and glycemic traits among representative species abundance levels in non-carriers (n=1,399). The participants were classified into three species abundance levels (low, middle and high) by tertiles. In each group, the associations of glycemic traits with alcohol consumption levels (0,1,2,3) were assessed by rank-based robust linear regression in which alcohol consumption level was treated as a continuous variable and covariates mentioned above were adjusted. The differences in associations of groups was assessed by testing the interaction between two continuous variables (abundance tertiles and alcohol consumption level) in the robust regression model. * denotes P-interaction <0.05 and ** denotes P-interaction <0.01.
